# Systemic lupus erythematosus complicated by diffuse alveolar haemorrhage: risk factors, therapy and survival

**DOI:** 10.1136/lupus-2015-000117

**Published:** 2015-09-23

**Authors:** Nayef M Kazzaz, Patrick Coit, Emily E Lewis, W Joseph McCune, Amr H Sawalha, Jason S Knight

**Affiliations:** Division of Rheumatology, Department of Internal Medicine, University of Michigan, Ann Arbor, Michigan, USA

**Keywords:** Diffuse alveolar hemorrhage, Thrombocytopenia, Antiphospholipid Syndrome

## Abstract

**Objectives:**

While diffuse alveolar haemorrhage (DAH) is recognised as a life-threatening complication of systemic lupus erythematosus (SLE), little is known about its risk factors and response to treatment. We describe 22 cases of DAH in a US lupus cohort of approximately 1000 patients, and compare them to 66 controls from the same outpatient cohort.

**Methods:**

We captured variables pertaining to diagnoses of SLE and secondary antiphospholipid syndrome (APS), and analysed them by univariate testing. Those variables with p values <0.05 were then further considered in a multivariate model. Kaplan-Meier curves were constructed for each group, and survival was analysed by Log-rank test.

**Results:**

Of the 22 patients with DAH, 59% were diagnosed with DAH within 5 years of lupus diagnosis. By univariate testing, several manifestations of SLE and APS were more common in patients with DAH, including history of thrombocytopenia, cardiac valve disease, low C3, leucopenia, neuropsychiatric features, haemolysis, arterial thrombosis, lupus anticoagulant, secondary APS and low C4. On multivariate analysis, history of thrombocytopenia and low C3 were maintained as independent risk factors. Importantly, only two patients had platelet counts <50 000/µL at the time of the DAH episode, arguing that DAH was not simply a haemorrhagic complication of thrombocytopenia. All patients were treated with increased immunosuppression, including various combinations of corticosteroids, plasmapheresis, cyclophosphamide, rituximab and mycophenolate mofetil. Notably, all patients in the cohort survived their initial episode of DAH. While the patients with DAH did well in the short-term, their long-term survival was significantly worse than controls. Several of the deaths were attributable to thrombotic complications after recovering from DAH.

**Conclusions:**

To the best of our knowledge, this is the largest case–control study of lupus DAH to date. History of thrombocytopenia was strongly predictive of DAH (OR ∼40). A number of APS manifestations correlated with DAH by univariate analysis, and deserve further consideration in larger studies.

**Key messages**

- History of thrombocytopenia was a strong predictor of DAH in SLE; however, most patients did not have active thrombocytopenia at the time of DAH.
- Several manifestations of antiphospholipid syndrome correlated with DAH by univariate analysis and deserve further analysis in larger studies.
- While this cohort of DAH patients had very good acute outcomes, they had a long-term survival disadvantage when compared to lupus patients without DAH.

## Introduction

Diffuse alveolar haemorrhage (DAH) is considered a catastrophic complication of systemic lupus erythematosus (SLE). It can be acute, developing over hours, or subacute, emerging over several days.^1^ Depending on the study, DAH has been reported to complicate 2–5% of all cases of SLE.^2^ ^3^ DAH is typically defined by the presence of three major components: (1) signs (bronchoscopy with bloody return) or symptoms (dyspnoea, cough, haemoptysis) of pulmonary haemorrhage, (2) new drop in haemoglobin (typically 1.5–2 g/dL) and (3) new, diffuse infiltrates on chest imaging.^4–7^ Of note, most studies have found dyspnoea to be a far more common presenting symptom than haemoptysis, occurring in 74–100% of patients,^1^ ^6^ ^8–12^ as compared to 30–100% for haemoptysis.^1^ ^5^ ^6^ ^8–11^ ^13^ Lupus nephritis has frequently been linked to DAH, with evidence of active kidney disease in as many as 64–100% of patients with lupus DAH.^1^ ^5–8^ ^10^ ^11^ ^14^ ^15^ Hypocomplementemia^5–7^ ^11^ and cytopenias^5^ ^7^ ^8^ are other common DAH-associated features.

Regarding pathogenesis, lupus DAH is classically reported as a neutrophilic capillaritis with destruction of alveolar septae and infiltration of haemosiderin-laden macrophages.^14^ ^16^ ^17^ Some studies have additionally shown granular immune complex deposition in the alveolar septae,^14^ ^16^ ^17^ while a role for B cells has been suggested by murine models.^18^ Acute DAH mortality in SLE has ranged from 0% to 62%,^1^ ^5–11^ ^13^ ^15^ ^17^ with mortality mainly attributable to respiratory complications,^14^ thrombotic disease^15^ and central nervous system disease.^13^ As with most organ-threatening manifestations of SLE, cyclophosphamide has been the mainstay of therapy for DAH,^1^ ^14^ albeit in association with poor outcomes, which probably reflect its preferential use in the most critically ill patients.^1^ Other reported therapies include plasmapheresis,^19^ ^20^ extracorporeal membrane oxygenation (ECMO),^21^ ^22^ rituximab,^23–30^ mycophenolate mofetil,^31^ recombinant factor VII^32^ ^33^ and stem cell transplantation.^34^ The evidence to support any particular therapy is not strong.

Some, but not all, studies have also hinted at a link between antiphospholipid syndrome (APS) and DAH. In lupus DAH, anticardiolipin antibody prevalence has ranged from 12% to 25%,^6^ ^8^ ^9^ ^11^ with prevalence of the syndrome itself at 13.6%.^13^ In terms of primary APS, several case reports have characterised patients with antiphospholipid antibody-associated or APS-associated DAH,^35–38^ and have found radiological features essentially indistinguishable from those seen in SLE.^39^ Further, at least three primary APS case series have suggested an association between DAH and APS, with some of these cases actually demonstrating biopsy-proven capillaritis.^40–42^

We describe 22 cases of DAH in a US lupus cohort of approximately 1000 patients, and compare them to 66 controls from the same outpatient cohort. To the best of our knowledge, this is the largest case–control study of lupus DAH reported to date.

## Methods

### Patients

The Institutional Review Board of the University of Michigan approved this study, and all patients signed an informed consent form for review of their medical record. The Michigan Lupus Cohort currently includes around 1000 patients who meet the American College of Rheumatology classification criteria for SLE.^43^

We searched the cohort for the terms ‘DAH’, ‘diffuse alveolar haemorrhage’ and ‘pulmonary haemorrhage’. This search identified 36 patients whose charts were then carefully reviewed. Patients were considered to have DAH if they fulfilled three criteria. The first required the presence of at least one pulmonary sign or symptom, including dyspnoea, cough, haemoptysis, hypoxaemia, intubation or a bronchoscopy positive for bloody return. The second was haemoglobin drop of more than 1.5 g/dL over 48 h (as compared to baseline). The third was abnormal imaging suggestive of a new diffuse infiltrative process, typically either a chest X-ray or chest CT scan. Twenty-two patients met all three criteria and were considered to have DAH; these were the patients further considered. For each DAH case, three birth-year-matched lupus patients were identified from the same cohort. Therefore, the control group consisted of 66 lupus patients, none of whom had a history of DAH.

### Statistical analysis

Univariate conditional logistic regression was used to evaluate the relationship between DAH and the following predictor variables: sex (male, female), ethnicity (white, black, other), malar rash, the presence of any lupus rash, oral ulcers, alopecia, arthritis, serositis, nephritis, neuropsychological disturbance, Raynaud's phenomenon, leucopenia, haemolysis, thrombocytopenia, low C3, low C4, antinuclear antibodies, anti-dsDNA (double-stranded DNA), anti-Ro (Sjögren's syndrome A, SSA), anti-La (Sjögren's syndrome B, SSB), anti-Sm antibodies, anti-ribonucleoprotein (RNP), APS, arterial thrombosis, venous thrombosis, pregnancy loss, cardiac valve involvement, livedo reticularis, seizures, anti-ß_2_GPI (β-2 glycoprotein I) IgG, anti-ß_2_GPI IgA, anti-ß_2_GPI IgM, anti-cardiolipin IgG, anti-cardiolipin IgM and lupus anticoagulant.

Predictor variables were ranked based on the p value for the likelihood ratio χ^2^ test, as well as the OR. A multivariate conditional logistic regression model was built in a stepwise manner. Starting with the highest ranking variable, the next highest ranked variable was added to the equation. The Akaike information criterion (AIC) of the reduced and expanded models and the Wald p values of the predictor variables were compared. A variable was retained in the model if the AIC of the larger model was less than the AIC of the previous model and each variable had a p value <0.05. If the variable was rejected, the next ranking variable was introduced and the model was retested. Conditional logistical regression statistical analysis was conducted using SAS V.9.4.

For survival analysis, Kaplan-Meier curves were constructed for each group (DAH vs control), and survival was compared by Log-rank test.

## Results

### DAH in SLE

**Figure 1:**
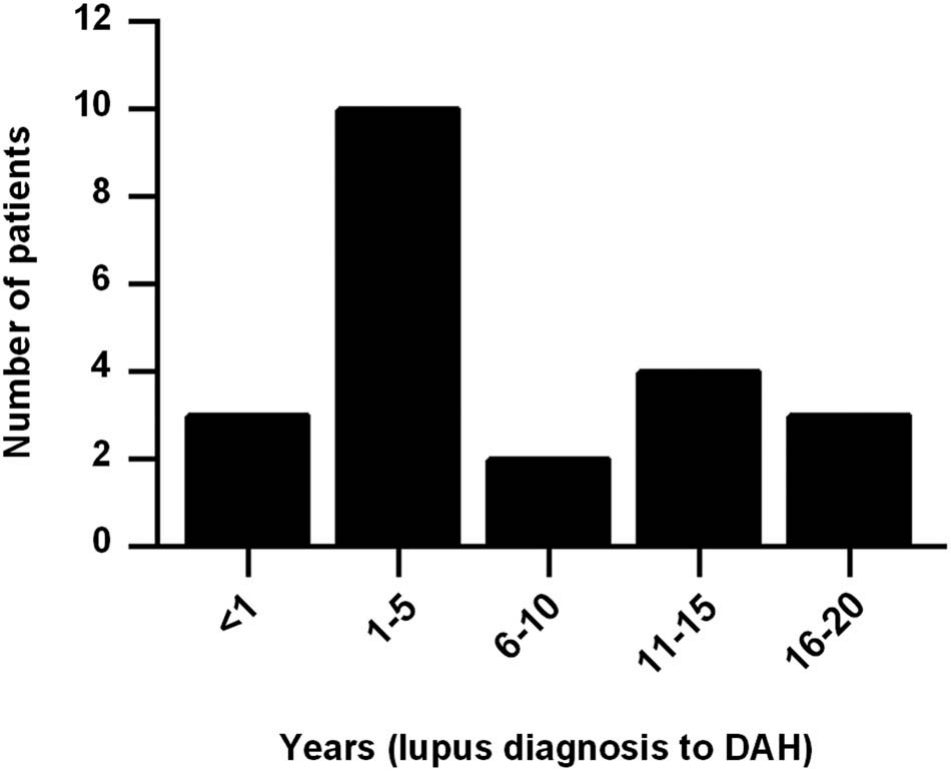
Interval between lupus diagnosis and first episode of diffuse alveolar haemorrhage (DAH).

We identified 22 cases of DAH in our lupus cohort, all of whom fulfilled the three criteria outlined above. Average ages at lupus and DAH diagnoses were 30 and 37 years old, respectively. DAH most commonly presented within 5 years of lupus diagnosis (figure 1). Most patients identified themselves as Caucasian or white (16/22, 73%). Two patients identified themselves as African-American or black, with the remaining patients identifying as Hispanic, Arab or Asian; the race of a single patient could not be determined from the medical record. Interestingly, and similar to prior studies,^1^ ^5^ ^6^ ^8–11^ ^13^ only 27% of patients had haemoptysis as a presenting symptom (table 1). Not surprisingly, DAH was often associated with worsening hypocomplementemia from baseline (75%), as well as increased anti-dsDNA levels from baseline (56%). Eight patients (36%) were diagnosed with nephritis concurrent to their DAH episode; four of these patients were new diagnoses, and four cases represented reactivation of dormant disease. Average systemic lupus erythematosus disease activity index (SLEDAI)-2K score at presentation was 11.4.^44^

**Table 1:** Diffuse alveolar haemorrhage presentation

Active features	Frequency	Percentage
Dyspnoea	14/22	(64)
Hypoxaemia*	12/22	(55)
Cough (no haemoptysis)	9/22	(41)
Haemoptysis	6/22	(27)
BAL		
Bloody return	8/14	(57)
Haemosiderin-laden MΦ	2/14	(14)
SLEDAI-2K score†	11.4±7.3	
Any lupus rash	5/22	(23)
Arthritis	4/22	(23)
Serositis	7/22	(32)
Nephritis	8/22	(36)
Neuropsychiatric	2/22	(9)
Leucopenia	5/21	(23)
Haemolytic anaemia	2/22	(9)
Thrombocytopenia	5/22	(23)
Raynaud's	2/22	(9)
Hypocomplementemia	15/20	(75)
Down from baseline	12/16	(75)
Elevated anti-dsDNA	14/18	(78)
Up from baseline	9/16	(56)

*Nine patients required intubation.

†Mean and SD are presented.

BAL, bronchoalveolar lavage; dsDNA, double-stranded DNA; MΦ, macrophage; SLEDAI, systemic lupus erythematosus disease activity index 2000.

All DAH diagnoses were made in hospitalised patients. DAH was the primary indication for admission in 17 of 22 cases (77%), while the remainder developed DAH during hospitalisation for a different indication. Five patients were reportedly on warfarin at the time of the DAH episode, although only one had an elevated international normalised ration (INR) (3.5) at the time of diagnosis; the other four were significantly subtherapeutic (INR 1.1 or lower). Five pulmonary embolism (PE) protocol CT scans were obtained as part of a DAH work up, and all five were negative for PE. Active thrombocytopenia (platelets <100 000/µL) was present in 5 of 22 patients (23%), although only 2 of these patients had severe thrombocytopenia (platelets <50 000/µL). Eight patients had blood smears ordered to specifically evaluate for microangiopathy, and all eight were negative (including for the two patients who clinically had active haemolysis). Similar to other case series,^1^ ^4^ ^5^ ^12^ a variety of treatments were employed (table 2). Highlighting a trend in the approach to therapy, all patients treated with rituximab (3/3) and 80% of patients treated with mycophenolate mofetil (4/5) presented after 2007.

**Table 2:** Treatment of the first DAH episode

Treatment	Frequency (n=22)	Percentage
Pulse methylprednisolone	10	45
Prednisone 1 mg/kg/day	4	18
Increased prednisone	7	32
Intravenous immunoglobulin	3	14
Plasmapheresis	4	18
Cyclophosphamide (any)	9	41
Rituximab (any)	3	14
Cyclophosphamide+rituximab	2	9
Mycophenolate mofetil	5	23

DAH, diffuse alveolar haemorrhage.

### Analysis of DAH-associated features

By univariate analysis, history of several lupus-associated and APS-associated features were more common in patients with DAH than controls (table 3). However, when a multivariate model was constructed, only two features—history of thrombocytopenia (OR=36) and history of low complement C3 (OR=19)—were maintained as independent risk factors.

**Table 3:** Comparison between DAH and control groups (univariate analysis)

Historical features	DAH (n=22)	Control (n=66)	OR (CI)	p Value
Malar rash	8/22	33/66	0.61 (0.24 to 1.56)	0.3
Any lupus rash	17/22	44/66	1.65 (0.56 to 4.93)	0.35
Oral ulcers	6/22	16/66	1.16 (0.41 to 3.28)	0.79
Alopecia	5/22	10/66	1.6 (0.50 to 5.09)	0.44
Arthritis	19/22	63/66	0.18 (0.02 to 1.82)	0.11
Serositis	14/22	33/66	2.07 (0.67 to 6.36)	0.20
Nephritis	14/22	28/66	2.52 (0.89 to 7.16)	0.07
Neuropsychiatric	10/22	9/66	9.20 (1.94 to 43.59)	**0.001**
Raynaud's	5/22	24/66	0.54 (0.18 to 1.59)	0.24
Haemolysis	12/22	10/66	5.85 (2.01 to 17.03)	**0.001**
Thrombocytopenia*	19/22	10/66	43.2 (5.72 to 326.40)	**<0.0001**
Leucopenia	21/22	39/66	13.43 (1.74 to 103.79)	**0.0004**
Low C3*	20/22	29/66	19.26 (2.49 to 148.72)	**<0.0001**
Low C4	16/22	31/66	3.32 (1.05 to 10.54)	**0.03**
ANA	21/22	58/64	2.27 (0.26 to 20.06)	0.43
Anti-dsDNA	18/22	41/65	2.45 (0.77 to 7.77)	0.11
Anti-SSA	7/21	27/64	0.65 (0.21 to 1.98)	0.44
Anti-SSB	3/21	13/63	0.70 (0.19 to 2.64)	0.6
Anti-Sm	8/21	16/63	2.59 (0.79 to 8.45)	0.11
Anti-RNP	8/21	22/63	1.23 (0.44 to 3.44)	0.69
Arterial thrombosis	7/22	4/66	6.42 (1.65 to 25.09)	**0.01**
Venous thrombosis	7/22	10/66	2.62 (0.84 to 8.15)	0.1
Pregnancy loss	3/18	4/60	2.81 (0.56 to 13.96)	0.22
Cardiac valve disease	8/22	2/66	22.09 (2.75 to 177.67)	**<0.0001**
Livedo reticularis	2/22	5/66	1.2 (0.23 to 6.19)	0.83
Seizures	4/22	4/66	4.37 (0.77 to 24.92)	0.09
Anti-β_2_GPI IgG	2/21	9/55	0.59 (0.11 to 3.05)	0.52
Anti-β_2_GPI IgA	3/21	11/55	0.70 (0.18 to 2.80)	0.61
Anti-β_2_GPI IgM	1/21	3/55	1.0 (0.10 to 9.61)	1
Cardiolipin IgG	7/22	16/63	1.35 (0.46 to 3.98)	0.58
Cardiolipin IgM	2/22	4/62	1.39 (0.26 to 7.66)	0.71
Lupus anticoagulant	9/20	7/60	5.36 (1.63 to 17.68)	**0.004**
APS	6/22	6/66	4.37 (1.06 to 18.11)	**0.04**

*Maintained as statistically significant risk factors in a multivariate model. Statistically-significant p values (p<0.05) are presented in bold.

ANA, antinuclear antibody; APS, antiphospholipid syndrome; DAH, diffuse alveolar haemorrhage; dsDNA, double-stranded DNA; RNP, ribonucleoprotein; SSA, Sjögren's syndrome A; SSB, Sjögren's syndrome B; β_2_GPI, β-2 glycoprotein I.

### Mortality and recurrence

**Figure 2:**
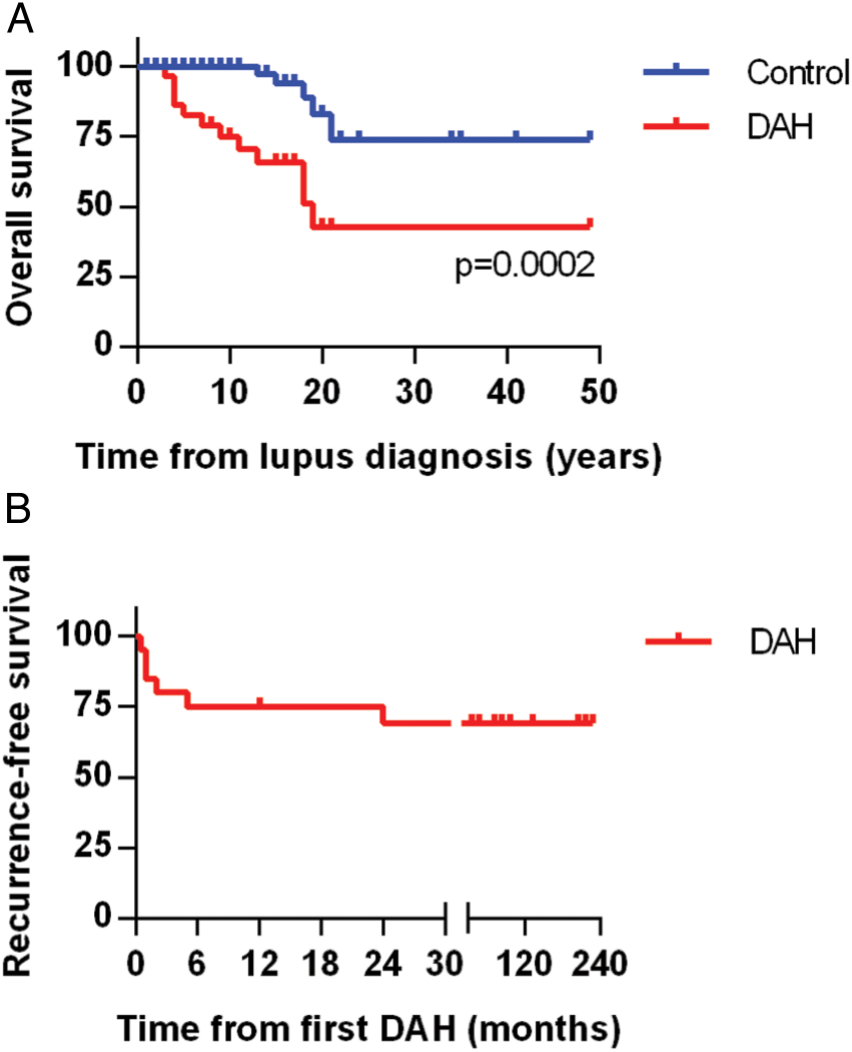
Overall and recurrence-free survival. (A) Kaplan-Meier curve comparing mortality of the lupus diffuse alveolar haemorrhage (DAH) group and the lupus controls. Lupus diagnosis is the point of reference and is set as time zero. (B) Kaplan-Meier curve demonstrating DAH recurrence-free survival in the lupus DAH group. Notably, all but one recurrence was within 6 months of the original episode.

Although prior studies have shown significant acute mortality from DAH episodes in SLE, this was not apparent in our study, regardless of which treatment was employed. Notably, all 22 patients survived their DAH episode and were discharged from the hospital. Nevertheless, long-term mortality in our DAH cohort was significantly higher when compared to the control group (figure 2A). Causes of death in patients with DAH included stroke (2 patients), infectious complications (2), neurological decline (1) and unknown (1). DAH recurred in 6 of 22 patients (27%), typically within 6 months of the original presentation (figure 2B).

## Discussion

To the best of our knowledge, we present the largest case–control study of lupus DAH reported to date. There were similarities and differences between our findings and those in the work of others. For example, similar to previous studies, we found that dyspnoea was a more common presentation than haemoptysis.^1^ ^4–6^ ^8–13^ While active nephritis was often associated with DAH in our study (36% of patients), the percentage was somewhat lower than what has been described by others.^1^ ^5–8^ ^10^ ^11^ ^14^ ^15^ In comparison to the other large case series from Asia and South America,^4–7^ we describe a US lupus population that is predominantly of European descent. This difference in ethnicity may have contributed to the lower percentage of nephritis.^45–47^ It should also be pointed out that kidney biopsies were typically only obtained if the clinician felt it would change clinical management. Given the degree of serological activity that was present in these patients (75% had decreased complement levels from baseline), it is reasonable to assume that several additional patients had early or subclinical nephritis that may have been effectively treated by the potent immunosuppression employed for DAH.^48^ ^49^

Also notable is that all 22 patients survived their first DAH episode; further, although 6 patients had recurrence, none resulted in death. In contrast, prior studies have shown acute mortality as high as 62%.^1^ ^5–9^ ^11^ ^13^ ^17^ Ours is a predominantly outpatient cohort, with the majority of patients recruited and enrolled during a visit to outpatient clinic. These patients were carefully followed and monitored, and it is possible that this attention resulted in earlier diagnosis as well as more aggressive treatment. It is also notable that the majority of our patients (14/22, 64%) were diagnosed after 2007; it is therefore conceivable that newer therapeutic agents such as mycophenolate mofetil and rituximab contributed to better outcomes and more durable induction of remission. Finally, it should again be pointed out that this is the first time a lupus population predominantly of European descent has been considered in a large DAH case series; as such, it is possible that this is a population predisposed to better outcomes.^1^ ^15^

There are no randomised clinical trials to inform our management of patients with lupus DAH. Retrospective studies have shown a survival disadvantage with cyclophosphamide, although it has been speculated that this is the result of cyclophosphamide's preferential use in the most ill patients.^1^ ^4^ Such studies have also been unable to show a clear survival benefit of either intravenous immunoglobulin or plasmapheresis in lupus DAH, although these treatments are commonly employed in severe cases (including at our centre).^1^ ^5^ ^12^ Rituximab is another emerging treatment, although again without either retrospective or prospective data to support its use.^4^ In the absence of clear guidance from the literature, the approach to treatment remains individualised.

**Figure 3:**
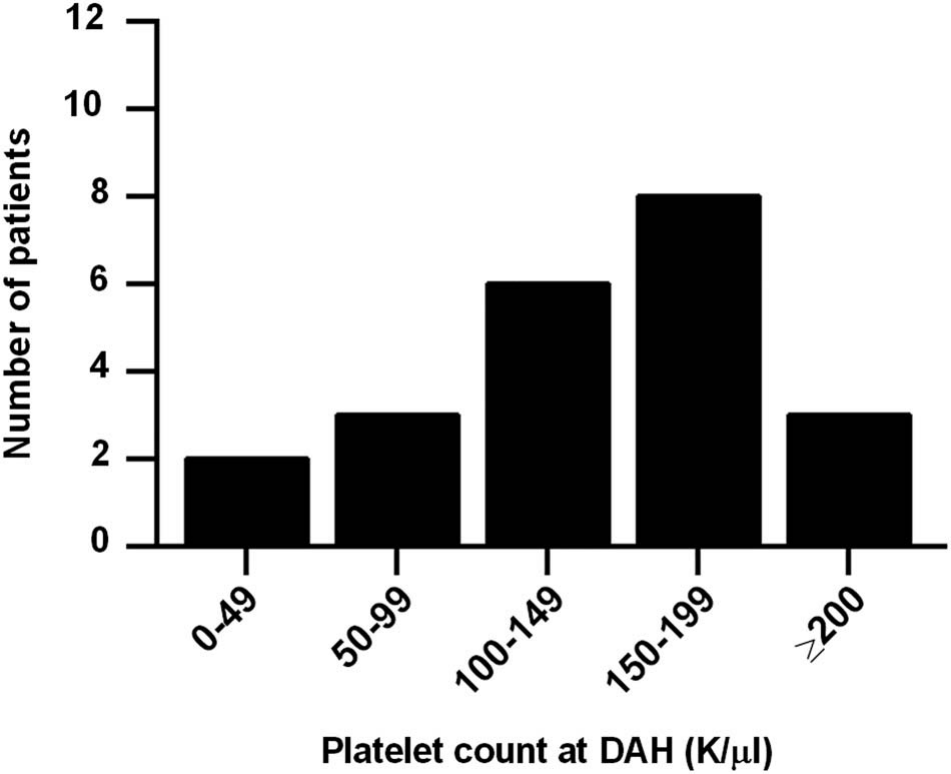
Platelet count at the time of first episode of diffuse alveolar haemorrhage (DAH). While only five patients had a platelet count <100 000/µL at the time of the DAH episode, 19 of 22 had such a count as part of their lupus history.

Of interest, history of both leucopenia and thrombocytopenia were strongly associated with DAH, with history of thrombocytopenia, in particular, being the strongest individual predictor of DAH in our study. In fact, after multivariate modelling, all risk was explained by just two factors: history of thrombocytopenia and history of low complement C3. We initially considered the possibility that DAH might be explained by the bleeding diathesis of thrombocytopenia itself, but this seems unlikely as only five patients presented with platelet counts below 100 000/µL and only two with platelets <50 000/µL (figure 3). For the remainder, the thrombocytopenia (<100 000/µL) was part of their medical history, but not active at the time of DAH. It should also be pointed out that thrombocytopenia, similar to nephritis and neuropsychiatric disease, is a well-known predictor of worse prognosis in lupus.^50–53^ Platelets are known mediators of inflammation, for example by engaging neutrophils,^54^ ^55^ and may be depleted systemically as they concentrate at sites of local inflammation. It has also been suggested that there are immunological difference between the antiplatelet immune response in lupus, as compared to idiopathic immune thrombocytopenia,^56^ although it is unclear what implications this may have for other aspects and manifestations of SLE.

Although thrombocytopenia ultimately dominated the multivariate model, we did note that a number of APS features, including lupus anticoagulant, arterial thrombosis, valve disease and the syndrome itself, were associated with DAH by univariate analysis. Since APS can also drive thrombocytopenia,^57^ these relationships are complicated and will need to be clarified in larger cohorts that ideally also include patients with primary APS. It is noteworthy that in our series, two of the patients with DAH ultimately died of thrombotic strokes, while a third had a progressive neurological decline related to refractory seizures that may have been driven by APS. While the relationship between lupus DAH and APS has not been thoroughly explored in the literature, there are many reports and series describing DAH as a complication of primary APS.^35–38^ Again, given the likely complex pathophysiology, these relationships will need to be tracked in larger studies.

Our study has several limitations. The rarity and acuity of DAH limit our ability to obtain prospective data, although this would obviously be the preferred methodology. As highlighted above, this is also a cohort that was primarily established in our outpatient clinics. It is therefore possible that we missed patients, especially those with poor and acute outcomes, who received their outpatient care elsewhere and were transferred to our centre for inpatient management. These data should therefore be interpreted in that light. Finally, because the acute outcomes were so good, we were not able to comment on the response to different types of treatments, which had been a goal when we devised the study.

In summary, using a case–control methodology, we showed a significant association between history of thrombocytopenia and lupus DAH. In fact, an amazing 19 of 22 patients (86%) had a history of platelets <100 000/µL at some point during their lupus course, and we would suggest that DAH should be featured prominently in the differential diagnosis when such patients present with pulmonary complaints. This is especially true as haemoptysis is only present in about one-in-four presentations, and therefore the index of suspicion must be high to establish the diagnosis of DAH. Further, although thrombocytopenia dominated the multivariate model, our univariate analysis suggests that secondary APS (similar to primary APS) may be a risk factor for DAH. While we await with interest larger multicentre considerations of this rare, but dangerous, lupus complication, we were encouraged to find that a carefully monitored cohort can fair well with DAH, especially acutely.
